# Postoperative Delirium After Pheochromocytoma Resection: An Exploratory Retrospective Single‐Center Cohort Study

**DOI:** 10.1155/anrp/8487117

**Published:** 2026-11-15

**Authors:** Jamal Ouachaou, Aicha Driouich, Mohammed Sidayne, Fatima Zahra Elkhattab, Youssef Zarrouki

**Affiliations:** ^1^ Anesthesiology and Intensive Care Mohammed VI University Hospital of Marrakech Marrakesh Morocco chumarrakech.ma; ^2^ Faculty of Medicine and Pharmacy Cadi Ayyad University Marrakesh Morocco uca.ma

**Keywords:** adrenalectomy, CAM-ICU, catecholamine withdrawal, hemodynamic instability, pheochromocytoma, postoperative delirium

## Abstract

**Background:**

Perioperative management of pheochromocytoma has traditionally focused on cardiovascular stability, while acute postoperative neurocognitive outcomes remain insufficiently documented. This study aimed to evaluate the incidence, associated clinical factors, and postoperative outcomes of probable postoperative delirium (POD) following pheochromocytoma resection in an exploratory single‐center cohort.

**Methods:**

We conducted a retrospective, single‐center observational cohort study of 30 consecutive adult patients who underwent adrenalectomy for pheochromocytoma between January 2022 and May 2026, diagnosed preoperatively on clinical, biochemical, and radiological grounds and confirmed by postoperative histopathological examination. Probable POD was identified through retrospective medical record auditing combined with documented Confusion Assessment Method for the Intensive Care Unit (CAM‐ICU) evaluations performed throughout the ICU/PACU stay. We compared continuous variables using Student’s *t*‐test (with Welch’s correction when appropriate) or Mann–Whitney *U* test and categorical data using Fisher’s exact test.

**Results:**

The overall incidence of retrospectively identified probable POD was 33.3% (*n* = 10). Clinical phenotypes included psychomotor agitation (*n* = 6), hallucinations/confusion (*n* = 3), and hypoactive somnolence (*n* = 1). In unadjusted exploratory analyses, POD was significantly associated with older age (56.0 ± 9.3 vs. 47.4 ± 10.3 years, *p* = 0.034), open laparotomy (90.0% vs. 25.0%, *p* = 0.001), higher baseline ASA score (ASA III: 60.0% vs. 10.0%, *p* = 0.010), larger tumor size (44.9 ± 6.0 vs. 39.3 ± 7.5 mm, *p* = 0.048), and deeper postresection diastolic hypotension (nadir DBP: 55.7 ± 5.4 vs. 60.4 ± 3.7 mmHg, *p* = 0.027). Patients experiencing POD had a longer median ICU stay (2.5 vs. 1.0 days, *p* = 0.011). Total in‐hospital mortality was 6.7% (*n* = 2), occurring exclusively in nondelirious patients experiencing refractory vasoplegic shock.

**Conclusion:**

Probable POD occurred in one‐third of patients following pheochromocytoma resection and was associated with open surgery, older age, tumor size, and postresection diastolic hypotension in exploratory unadjusted comparisons. Standardized prospective screening and targeted hemodynamic management warrant further investigation in larger prospective studies.

## 1. Introduction

Pheochromocytoma is a rare neuroendocrine tumor arising from chromaffin cells of the adrenal medulla, characterized by autonomous, paroxysmal secretion of catecholamines, specifically epinephrine and norepinephrine [1]. Historically, surgical resection carried prohibitive perioperative mortality rates of 25%–40%, predominantly driven by acute intraoperative hypertensive crises and severe postresection circulatory collapse [2, 3]. Over recent decades, optimized preoperative alpha‐adrenergic blockade and modern anesthetic strategies have markedly reduced perioperative mortality to under 5% in specialized surgical centers, though postoperative morbidity remains significant [4].

While cardiovascular and renal complications such as refractory vasoplegic shock and acute kidney injury have been extensively investigated, acute postoperative neurocognitive alterations remain insufficiently documented in this patient population [5]. Postoperative delirium (POD)—an acute disturbance of attention, awareness, and cognition that fluctuates in severity—is a well‐established predictor of adverse clinical outcomes, prolonged hospitalization, and increased healthcare costs in general surgical and intensive care unit (ICU) populations.

The acute endocrine dynamics of pheochromocytoma surgery subject the central nervous system to unique homeostatic challenges. Intraoperative tumor manipulation can trigger surge‐like release of catecholamines, whereas ligation of the primary adrenal drainage leads to an abrupt withdrawal of circulating catecholamines, frequently precipitating sudden systemic vasoplegia and transient cerebral hypoperfusion [6]. It is hypothesized that these rapid endocrine and hemodynamic fluctuations, alongside surgical trauma and perioperative neuroinflammation, may disrupt central neurotransmitter pathways and cerebral microcirculation, directly contributing to acute cognitive dysfunction [6].

To explore this underreported clinical entity, we conducted a retrospective cohort study of 30 consecutive patients undergoing pheochromocytoma resection at a tertiary referral center. The primary objective was to estimate the incidence of retrospectively identified probable POD and evaluate its univariate clinical associations. We hypothesized that lower postresection diastolic blood pressure (DBP), open surgical resection, and increased tumor burden would be associated with a higher likelihood of POD.

## 2. Materials and Methods

### 2.1. Study Design and Ethics

We performed an exploratory, retrospective, single‐center analytical cohort study at the Errazi University Hospital Center (Mohammed VI University Hospital) in Marrakech, Morocco. The study evaluated consecutive patients who underwent adrenalectomy for pheochromocytoma between January 2022 and May 2026, with the diagnosis confirmed by postoperative histopathological examination of the resected specimen. The Institutional Review Board and Ethics Committee of the Mohammed VI University Hospital Center formally approved the study protocol (Approval No. 88/2026). Given the retrospective observational design, the requirement for written informed consent was waived.

### 2.2. Participant Selection and Flow

Eligible participants were adult patients (≥ 18 years of age) who underwent elective surgical resection for pheochromocytoma during the study timeframe. The preoperative diagnosis was based on a combination of clinical presentation (e.g., paroxysmal or sustained hypertension, headache, sweating, palpitations, or incidentally discovered adrenal mass), biochemical findings (elevated 24‐h urinary or plasma metanephrines/normetanephrines), and radiological identification of an adrenal mass on CT or MRI. The diagnosis was subsequently confirmed postoperatively by histopathological examination of the resected specimen in the Department of Pathology. Only patients with histologically confirmed pheochromocytoma on the final pathology report were included. Exclusion criteria were defined as

1. Incomplete perioperative medical records;
2. Documented pre‐existing neurocognitive impairment or formal diagnosis of dementia;
3. Severe underlying psychiatric illness;
4. Emergency surgical procedures unrelated to elective pheochromocytoma resection.

During the study period, we identified 30 consecutive eligible patients; no eligible cases were excluded (Figure 1).

**FIGURE 1:**
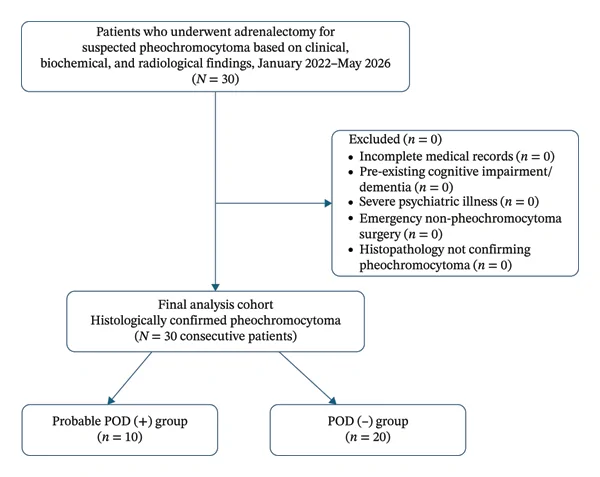
STROBE participant flow diagram.

### 2.3. POD Ascertainment

Because this study was retrospective, delirium status was identified using a standardized dual‐audit methodology combining contemporaneous nurse and physician chart reviews with documented Confusion Assessment Method for the Intensive Care Unit (CAM‐ICU) evaluations [7]. CAM‐ICU assessments were performed by ICU medical and nursing staff as part of routine clinical care and documented in the patients’ records throughout the entire ICU/PACU stay, from emergence from anesthesia until transfer to the surgical ward; assessment was therefore not restricted to a specific postoperative day. These documented assessments were retrospectively extracted for the present study. Patients were classified as having probable POD if they had either a positive CAM‐ICU assessment documented during ICU or PACU stay, OR clear, acute, and fluctuating narrative medical record documentation of cognitive disturbance (e.g., psychomotor agitation, visual/auditory hallucinations, disorientation, or transient hypoactive somnolence requiring heightened clinical surveillance or rescue antipsychotic administration with haloperidol) [8]. The primary observation window spanned from emergence from anesthesia until discharge from the ICU or PACU.

### 2.4. Intraoperative Management and Hemodynamic Definitions

All patients underwent preoperative stabilization using alpha‐adrenergic blockers (primarily phenoxybenzamine or doxazosin) adjusted to control resting blood pressure and restore intravascular volume. Continuous intravenous nicardipine infusion was administered intraoperatively to control hypertensive surges during tumor manipulation. We monitored intraoperative hemodynamics continuously via invasive arterial catheterization. We defined the minimal intraoperative DBP as the lowest DBP nadir recorded after adrenal vein ligation during the vasoplegic phase, before arterial line removal.

### 2.5. Statistical Analysis

Statistical analyses were performed using SPSS Version 26.0 (IBM Corp., Armonk, NY, USA). We assessed continuous variables for normality using visual inspection and the Shapiro–Wilk test. Normally distributed continuous variables are presented as mean ± standard deviation (SD) and were compared between POD (+) and POD (−) groups using the independent samples *t*‐test (with Welch’s correction applied when equal variances could not be assumed). Nonnormally distributed continuous variables (ventilation duration and ICU stay) are presented as median and interquartile range (IQR) and were compared using the Mann–Whitney *U* test. Categorical variables are expressed as counts and percentages (*n*, %) and were compared using Fisher’s exact test to account for sparse cell counts. Two‐tailed *p* values <0.05 were considered statistically significant. Due to the small number of outcome events (*n* = 10), multivariable logistic regression modeling was not performed to prevent model overfitting.

## 3. Results

### 3.1. Cohort Baseline Characteristics

A total of 30 consecutive patients met all eligibility criteria and were included in the final analysis. The mean age of the overall cohort was 50.3 ± 10.6 years (range: 28–67 years), with 13 males (43.3%) and 17 females (56.7%). Mean overall tumor diameter on CT imaging was 41.1 ± 7.5 mm. Overall mean minimal intraoperative DBP across the entire cohort was 58.8 ± 4.8 mmHg.

### 3.2. Incidence and Phenotypes of Delirium

Probable POD was identified in 10 out of 30 patients, yielding an estimated incidence of 33.3% (95%2009CI : 17.3%−52.8%) [9]. Clinical presentations among POD (+) cases were categorized as psychomotor agitation (*n* = 6, 60.0%), visual/auditory hallucinations and confusion (*n* = 3, 30.0%), and hypoactive somnolence (*n* = 1, 10.0%). Pharmacological intervention with haloperidol was required in 4 patients (40.0% of POD cases), while the remaining 6 cases resolved with supportive care within 24–48 h.

### 3.3. Exploratory Univariate Comparison: POD (+) vs. POD (−)

Comparative baseline, intraoperative, and outcome data between patients with POD (*n* = 10) and without POD (*n* = 20) are summarized in Table 1.

• Demographics and Surgical Factors: Patients in the POD (+) group were significantly older (56.0 ± 9.3 vs. 47.4 ± 10.3 years, *p* = 0.034) and had a higher proportion of ASA III physical status (60.0% vs. 10.0%, *p* = 0.010). Open laparotomy was performed in 90.0% (9/10) of POD (+) cases compared to 25.0% (5/20) of POD (−) cases (*p* = 0.001).
 Tumor Size and Hemodynamics: Mean tumor diameter was larger in the POD (+) group (44.9 ± 6.0 vs. 39.3 ± 7.5 mm, *p* = 0.048). Following adrenal vein ligation, patients who developed POD exhibited deeper diastolic hypotension, with a lower minimal DBP nadir (55.7 ± 5.4 vs. 60.4 ± 3.7 mmHg, *p* = 0.027, Welch’s *t*‐test; Student’s *t*‐test *p* = 0.009). Maximal intraoperative systolic blood pressure during tumor manipulation was also higher in the POD (+) group (221.4 ± 10.2 vs. 212.0 ± 12.1 mmHg, *p* = 0.042).
• Postoperative Complications and Outcomes: Acute kidney injury occurred in 1 patient (10.0%) in the POD (+) group and 3 patients (15.0%) in the POD (−) group (*p* = 1.000). Patients experiencing POD had a significantly longer median ICU stay (2.5 days [IQR: 2.0 − 3.0] vs. 1.0 day [IQR: 1.0 − 2.0], *p* = 0.011). Median ventilation duration did not differ significantly between groups (27.0 h [IQR: 22.5 − 34.0] vs. 19.5 h [IQR: 16.0 − 24.8], *p* = 0.052). Total in‐hospital mortality was 6.7% (*n* = 2), caused by refractory vasoplegic shock in ASA III patients, both occurring in the POD (−) group.

**TABLE 1:** Baseline characteristics, intraoperative parameters, and clinical outcomes of the cohort according to postoperative delirium status.

Parameter	Total cohort (*N* = 30)	POD (+) group (*n* = 10)	POD (−) group (*n* = 20)	*p* value	Statistical test
Age (years), mean ± SD	50.3 ± 10.6	56.0 ± 9.3	47.4 ± 10.3	**0.034**	Student’s *t*‐test
Male sex, *n* (%)	13 (43.3%)	3 (30.0%)	10 (50.0%)	0.428	Fisher’s exact
ASA Class III, *n* (%)	8 (26.7%)	6 (60.0%)	2 (10.0%)	**0.010**	Fisher’s exact
Surgical approach (laparotomy), *n* (%)	14 (46.7%)	9 (90.0%)	5 (25.0%)	**0.001**	Fisher’s exact
Tumor size (mm), mean ± SD	41.1 ± 7.5	44.9 ± 6.0	39.3 ± 7.5	**0.048**	Student’s *t*‐test
Maximal intraop SBP (mmHg), mean ± SD	215.1 ± 12.2	221.4 ± 10.2	212.0 ± 12.1	**0.042**	Student’s *t*‐test
Minimal intraop DBP (mmHg), mean ± SD	58.8 ± 4.8	55.7 ± 5.4	60.4 ± 3.7	**0.027**	Welch’s *t*‐test^∗^
Acute kidney injury, *n* (%)	4 (13.3%)	1 (10.0%)	3 (15.0%)	1.000	Fisher’s exact
Ventilation duration (hours), median (IQR)	20.0 (16.0–27.8)	27.0 (22.5–34.0)	19.5 (16.0–24.8)	0.052	Mann–Whitney *U*
ICU length of stay (days), median (IQR)	1.0 (1.0–2.0)	2.5 (2.0–3.0)	1.0 (1.0–2.0)	**0.011**	Mann–Whitney *U*
In‐hospital mortality, *n* (%)	2 (6.7%)	0 (0.0%)	2 (10.0%)	0.538	Fisher’s exact

*Note:* Statistically significant values (*p* < 0.05) are highlighted in bold. POD: postoperative delirium; IQR: interquartile range. We compared continuous variables using Student’s *t*‐test, or Welch’s *t*‐test when we could not assume equal variances.

Abbreviations: ASA, American Society of Anesthesiologists; DBP, diastolic blood pressure; ICU, intensive care unit; SBP, systolic blood pressure; SD, standard deviation.

^∗^Student’s *t*‐test (equal variances assumed) yields *p* = 0.009.

## 4. Discussion

In this exploratory single‐center retrospective cohort, probable POD was identified in 33.3% of patients undergoing pheochromocytoma resection. In univariate exploratory analyses, POD was associated with older age, open surgical approach, higher ASA physical status, larger tumor diameter, and lower minimal postresection DBP.

The observed delirium rate of 33.3% highlights that acute neurocognitive disturbances represent a relevant postoperative issue in pheochromocytoma surgery [9]. The pathogenesis of POD in this setting is likely multifactorial. Pheochromocytoma resection induces abrupt neuroendocrine changes: Surgical handling of the tumor produces intense catecholaminergic peaks that subject the blood–brain barrier to extreme hydrostatic pressure [10]. Conversely, adrenal vein ligation terminates tumor secretion, precipitating abrupt catecholamine withdrawal and severe vasoplegia [11]. In our cohort, patients developing POD recorded significantly lower minimal intraoperative DBP values (55.7 ± 5.4 mmHg), causing a transient but critical reduction in cerebral perfusion pressure [12].

Surgical approach demonstrated a strong association with delirium, as 90% of delirious patients underwent open laparotomy compared to 25% in nondelirious patients (*p* = 0.001). However, this unadjusted association must be interpreted cautiously due to confounding by indication: Open surgery was preferentially performed in patients with larger tumors (44.9 mm mean diameter in POD+) or complex anatomical features. Open laparotomy involves greater tissue disruption, prolonged surgical duration, higher postoperative pain levels, and increased systemic cytokine release—factors known to promote neuroinflammation and blood–brain barrier disruption [13, 14].

Regarding postoperative outcomes, patients experiencing POD had a modestly longer median ICU stay (2.5 vs. 1.0 days, *p* = 0.011) [15]. However, POD was not associated with increased mortality or prolonged mechanical ventilation in this series. Early detection and management of POD using validated tools such as the CAM‐ICU remain essential [16]. Major adverse events, including the cohort’s two deaths from refractory vasoplegic shock and a case requiring tracheostomy, occurred in nondelirious patients with severe underlying cardiovascular instability.

### 4.1. Study Limitations

This study has several important limitations:

1. Delirium status was determined retrospectively, which carries a risk of underdiagnosis or misclassification compared to continuous prospective screening. In particular, the narrative criterion relied on retrospective interpretation of medical and nursing records, which may not have been uniformly maintained; underdetection of hypoactive delirium cannot be excluded, which is why delirium is reported as “probable” throughout the manuscript.
2. With 30 patients and 10 delirium events, multivariable adjustment could not be performed without severe risk of overfitting. Observed associations cannot establish independent causality.
3. DBP was recorded as a discrete minimal nadir rather than continuous area‐under‐the‐curve hypotension exposure.

## 5. Conclusion

In summary, retrospectively identified probable POD occurred in 33.3% of patients following pheochromocytoma resection and was associated with open surgical approach, older age, larger tumor size, and deeper postresection diastolic hypotension in exploratory unadjusted analyses. Future prospective studies utilizing standardized daily CAM‐ICU protocols and continuous hemodynamic modeling are warranted to clarify independent risk factors and optimize perioperative neuroprotective strategies.

## Author Contributions

Jamal Ouachaou: conceptualization, methodology, data curation, software, formal analysis, and writing–original draft preparation. Aicha Driouich: data curation, software, formal analysis, and writing. Mohammed Sidayne: methodology, data curation, software, and formal analysis. Fatima Zahra Elkhattab: methodology and data curation. Youssef Zarrouki: supervision, validation, project administration, and writing–review and editing.

## Funding

This research did not receive any specific grant from funding agencies in the public, commercial, or not‐for‐profit sectors.

## Ethics Statement

This study was conducted in accordance with the Declaration of Helsinki and received formal institutional approval from the Ethics Committee and Institutional Review Board of the Mohammed VI University Hospital Center (CHU Mohammed VI, Marrakech, Morocco; Approval No. 88/2026). Due to the retrospective nature of this observational cohort study, the requirement for written informed consent was waived by the institutional review board.

## Conflicts of Interest

The authors declare no conflicts of interest.

## Supporting Information

Additional supporting information can be found online in the Supporting Information section.

## Supporting information


**Supporting Information** STROBE checklist.

## Data Availability

The clinical datasets used and analyzed during the current study are available from the corresponding author on reasonable request.
