# Supplementary material for: Postoperative Delirium After Pheochromocytoma Resection: An Exploratory Retrospective Single‐Center Cohort Study
Source: Anesthesiol Res Pract. 2026 Nov 15;2026:8487117. doi: 10.1155/anrp/8487117 (PMC13770305; doi:10.1155/anrp/8487117)
Supplement: Supplementary file 1 [file ANRP-2026-8487117-s001.docx]

**STROBE Statement**

| Item No. | Recommandations | Reported on Page No. |
| --- | --- | --- |
| TITLE and ABSTRACT | | |
| 1a | Title: Indicate the study’s design with a commonly used term in the title or the abstract. | Page 1 |
| 1b | Abstract: Provide in the abstract an informative and balanced summary of what was done and what was found. | Page 2 |
| INTRODUCTION | | |
| 2 | Background / Rationale: Explain the scientific background and rationale for the investigation being reported. | Page 3 |
| 3 | Objectives: State specific objectives, including any prespecified hypotheses. | Page 3 |
| METHODS | | |
| 4 | Study Design: Present key elements of study design early in the paper. | Page 4 |
| 5 | Setting: Describe the setting, locations, and relevant dates, including periods of recruitment, exposure, follow-up, and data collection. | Page 4 |
| 6a | Eligibility Criteria: Give the eligibility criteria, and the sources and methods of selection of participants. Describe methods of follow-up. | Page 4 |
| 7 | Variables: Clearly define all outcomes, exposures, predictors, potential confounders, and effect modifiers. Give diagnostic criteria, if applicable. | Page 4–5 |
| 8 | Data Sources / Measurement: For each variable of interest, give sources of data and details of methods of assessment (measurement). Describe comparability of assessment methods if there is more than one group. | Page 4–5 |
| 9 | Bias: Describe any efforts to address potential sources of bias. | Page 5, Page 8 |
| 10 | Study Size: Explain how the study size was arrived at. | Page 4 |
| 11 | Quantitative Variables: Explain how quantitative variables were handled in the analyses. If applicable, describe which groupings were chosen and why. | Page 5 |
| 12a | Statistical Methods: Describe all statistical methods, including those used to control for confounding. | Page 5 |
| 12b | Statistical Methods: Describe any methods used to examine subgroups and interactions. | Page 5 |
| 12c | Statistical Methods: Explain how missing data were addressed. | Page 4 |
| 12d | Statistical Methods: If applicable, explain how loss to follow-up was addressed. | Page 4 |
| 12e | Statistical Methods: Describe any sensitivity analyses. | N/A (Exploratory study) |
| RESULTS | | |
| 13a | Participants: Report numbers of individuals at each stage of study—eg numbers potentially eligible, examined for eligibility, confirmed eligible, included in the study, completing follow-up, and analysed. | Page 6, Figure 1 |
| 13b | Participants: Give reasons for non-participation at each stage. | Page 6, Figure 1 |
| 13c | Participants: Consider use of a flow diagram. | Figure 1 |
| 14a | Descriptive Data: Give characteristics of study participants (eg demographic, clinical, social) and information on exposures and potential confounders. | Page 6, Table 2 |
| 14b | Descriptive Data: Indicate number of participants with missing data for each variable of interest. | Page 4, Page 6 |
| 14c | Descriptive Data: Summarise follow-up time (eg, average and total amount). | Page 4 |
| 15 | Outcome Data: Report numbers of outcome events or summary measures over time. | Page 6, Table 2 |
| 16a | Main Results: Give unadjusted estimates and, if applicable, confounder-adjusted estimates and their precision (eg, 95% confidence interval). Make clear which confounders were adjusted for and why they were included. | Page 6, Table 2 |
| 16b | Main Results: Report category boundaries when continuous variables were categorised. | Page 5–6 |
| 16c | Main Results: If relevant, consider translating estimates of relative risk into absolute risk for a meaningful time period. | N/A |
| 17 | Other Analyses: Report other analyses done—eg analyses of subgroups and interactions, and sensitivity analyses. | Page 6 (Delirium Phenotypes) |
| DISCUSSION | | |
| 18 | Key Results: Summarise key results with reference to study objectives. | Page 8 |
| 19 | Limitations: Discuss limitations of the study, taking into account sources of potential bias or imprecision. Discuss both direction and magnitude of any potential bias. | Page 8 |
| 20 | Interpretation: Give a cautious overall interpretation of results considering objectives, limitations, multiplicity of analyses, results from similar studies, and other relevant evidence. | Page 8–9 |
| 21 | Generalisability: Discuss the generalisability (external validity) of the study results. | Page 8–9 |
| OTHER INFORMATION | | |
| 22 | Funding: Give the source of funding and the role of the funders for the present study and, if applicable, for the original study on which the present article is based. | Page 10 |
